# Break in Sedentary Behavior Reduces the Risk of Noncommunicable Diseases and Cardiometabolic Risk Factors among Workers in a Petroleum Company

**DOI:** 10.3390/ijerph14050501

**Published:** 2017-05-09

**Authors:** Chutima Jalayondeja, Wattana Jalayondeja, Keerin Mekhora, Petcharatana Bhuanantanondh, Asadang Dusadi-Isariyavong, Rujiret Upiriyasakul

**Affiliations:** 1 Faculty of Physical Therapy, Mahidol University, Salaya 73170, Thailand; wattana.jal@mahidol.ac.th (W.J.); keerin.mek@mahidol.ac.th (K.M.); petcharatana.bhu@mahidol.ac.th (P.B.); rujiret.upi@mahidol.ac.th (R.U.); 2 Medical and Occupational Health, PTT Exploration and Production Public Company Limited, Bangkok 10900, Thailand; asadangd@pttep.com

**Keywords:** noncommunicable diseases, physical activity, sedentary behavior, shiftwork, workplace

## Abstract

Although prolonged sitting appears as a novel risk factor related to health outcomes for all ages, its association needs to be replicated in occupational conditions. This study explored the associations between sedentary behavior and four noncommunicable diseases (NCDs) as well as two cardiometabolic risk factors (CMRFs) among workers in a petroleum company, Thailand. All workers were invited to complete the online self-report questionnaire. Sedentary behavior was measured as the amount of time sitting at work, during recreation, and while commuting. Out of 3365 workers contacted, 1133 (34%) participated. Prevalence of NCDs and CMRFs was 36% and was positively associated with sedentary behavior. After adjusting for age, BMI, and exercise, the risk of NCDs and CMRFs for sedentary office work was 40% greater compared with more active field work. Those who took a break without sitting more than twice a day and commuted by walking or cycling had less risk of NCDs and CMRFs. The total duration of sedentary behavior was 10 h/day, and two-thirds of that total was workplace sitting. This was significantly associated with NCDs and CMRFs (*p* < 0.001). Day-and-night rotating shiftwork was negatively associated with NCDs and CMRFs (*p* < 0.001). Sedentary behavior should be considered a health risk among workers. Hence, to promote a healthy lifestyle and safe workplace, organizations should encourage standing activities during break and physically active commutes, and have workers avoid prolonged sitting.

## 1. Introduction

Noncommunicable diseases (NCDs) are causes of morbidity and mortality worldwide. Four common NCDs, cardiovascular diseases (CVDs), diabetes mellitus (DM), chronic respiratory diseases (CRDs), and cancers (CAs), account for more than 38 million deaths globally [1]. Social, behavioral, and metabolic/physiological factors are three categories of preventable risk factors associated with NCDs. Hypertension and hyperlipidemia are the top 2 cardiometabolic risk factors (CMRFs) that account for 10 million deaths globally each year [2]. Physical activity (PA) is a behavioral factor that can prevent the risk of NCDs. Insufficient PA was reported among 31% of adults aged over 15 years. This sedentary behavior causes 3 million deaths each year worldwide [2,3]. Sitting and inclining activities were reported to be between 6 and 10 h a day in adults aged 20–59 years [4,5].

Four common NCDs caused 71% of deaths among Thai people age 15 years or older in 2014 [1]. The prevalence of insufficient PA was 19% in 2014 for all ages and up to 34% among children and adults in 2015 [6]. No progress in reducing insufficient PA in Thailand was made from 2008 to 2015, most probably due to the rapidly social change toward sedentary lifestyle. The average duration of sedentary behavior was reported as 13 h daily among the Thai population, which is greater than the average duration of sedentary behavior in some other countries [7,8].

The impact of urbanization on human lifestyle discourages participation in PA and motivates sedentary behavior [2]. Sedentary behavior includes sitting activities or expending very little energy (<1.5 metabolic equivalents or METs) at work, during recreation, and while commuting [4,9,10]. Recently, the amount of time using computers and daily sitting behavior has increased dramatically among workers and children. Owen and colleagues reported that 60% of adults had jobs that involved sitting at a computer and 90% of children sit with computers at school [4]. Additionally, household Internet availability has increased leading to longer periods of leisure time playing computer games and watching television (nearly 4 h daily) [4,11]. Many people use more than one hour of driving personal vehicles to commute to and from home to work [4]. The ownership of modern household possessions such as television (TV), computer, microwave and motorcycle promotes sitting behavior and is associated with either being overweight or obesity among women [12]. A preference for activities of less human energy expenditure or sedentary behavior is thus widespread nowadays.

The identification of sedentary behavior influencing overweight or obesity and disease occurrence has been reported in many studies [5,13,14,15,16,17]. Thorp and colleagues reviewed 46 prospective studies of sedentary behavior and reported that sedentary behavior, especially watching TV, was associated with health outcomes among children and adults [13]. The results demonstrated quantified evidence of a positive association between sitting to watch TV and disease mortality as well as weight gain/obesity in children. In contrast, findings reported a mixed association between watching TV and weight gain/obesity, disease incidences, and CMRFs in adults. The risk of disease morbidity and mortality was equal between men and women. Many studies have focused on leisure sedentary behavior, particularly watching TV, so exploring occupational and transportational sedentary activity is essential.

To examine the association between occupational sitting and health outcomes, van Uffelen and colleagues selected 43 papers [5]. Occupational sitting was defined as prolonged sitting or sedentary behavior at work compared with heavy labor or a high level of PA. They found conflicting results of the influence of occupational sitting on NCDs incidence including CVDs, DM, and cancers. Some studies strongly supported a positive association, while half of the research demonstrated none or negative associations. No difference was found in the evidence quality among the three directions of associations, and the prospective studies failed to confirm a causal relationship. A positive association was reported by 44% of study participants between occupational sitting time and CVDs, DM, and cancers after controlling for PA or exercise. Without controlling for the amount of PA, many studies reported positive or no association. Consequently, in future studies, the adjustment for PA and demographics variables should be considered in order to determine the association between sedentary behavior and health outcome among occupational workers.

The association between sedentary behavior and disease occurrence has been inconclusive, so many researchers have examined potential factors [5,13,14,15,16,17]. Possible confounders of the association need to be identified and controlled for to fully understand the relationship. Subsequently, recommendations need to be created to promote healthy lifestyles among those whose work is sedentary. Therefore, we aimed to investigate the association between sedentary behavior at work, during recreation, and while commuting and the occurrence of four common NCDs as well as two CMRFs among workers. We examined the association between types of activity and the total time spent sitting per day in relation to four common NCDs and two CMRFs among workers.

## 2. Materials and Methods

We conducted an online cross-sectional survey research among petroleum workers between 2013 and 2015. Their names, employee identification numbers, and positions were anonymous for personal information protection and confidentiality. We guaranteed that their participation in this study would have no consequences to their job security. This study was approved by the Mahidol University Institutional Review Board (COA. No.2013/109.3010).

An online self-reporting questionnaire on computer work-related exposure (OSCWE) consisted of five domains to survey health and related factors among computer workers [18]. The OSCWE was available and accessible, free at http://www.pt.mahidol.ac.th/ergo/index.php. The content validity and reliability test of the OSCWE were acceptable (Cronbach’s alpha 0.34–0.93) [18]. For data collection, the occupational health officers distributed the link to the web-based questionnaire via workers’ e-mails. Workers were asked to register to the OSCWE and to complete the survey within one month of registration. Incomplete self-administration questionnaires were reminded every two weeks to enhance the response rate. The questionnaire was submitted to the web master of the Faculty of Physical Therapy, Mahidol University, Thailand.

Subjects reported personal factors, health conditions, and sedentary behavior data. Personal factors were as follows: age comprised of four-decade groups (20–29, 30–39, 40–49, and 50–62 years), BMI categorized into four groups based on the WHO guideline for the Asian population [19] (BMI < 18.5, 18.5 ≤ BMI < 23, 23 ≤ BMI ≤ 27.5, BMI ≥ 27.5), weight change within two years (weight stable, weight gain, or weight loss), years of work experience (less than 3, 4–6, 7–10, and over 10 years), and frequency of exercise weekly (none, infrequent or once/week, sometimes, or 2–3 times/week, and often >3 times/week). These categorical personal factor variables were defined as confounding factors in this study.

Health condition information was collected regarding the underlying diseases. All workers must undergo an annual health check-up according to the Industrial Safety and Health Law. Twelve categories of health problems are listed. The worker could select all categories that pertain and answer open-ended question items of other problems for details. We were concerned with the disease occurrence of four common NCDs—cardiovascular diseases (CVDs), diabetes mellitus (DM), chronic respiratory diseases (CRDs), cancers (CAs)—and two cardiometabolic risk factors (CMRFs)—hypertension (HT) and hyperlipidemia. Subjects who reported at least one NCD, one CMRF, or a combination were defined as “having diseases”. Those who were healthy or had other conditions such as musculoskeletal disorders, gastrointestinal problems, and others were classified as “not having diseases”.

According to previous studies [4,9,10], we used the operational definition of sedentary behavior as occupational sitting for work with computer/document/meeting, sitting or reclining activities for playing computer/tablet/phone at home (not for work), and sitting or standing activities while commuting using passive modes (personal car, public transportation, employee shuttle bus, or motorcycle). We identified the amount of sedentary behavior based on the type of activity and the duration (hour/day) at work, during recreation, and while commuting. Work consisted of job description, shift work, and breaks taken while at work. Job descriptions were classified as office or field work. Office workers consisted of computer workers, managers, and secretaries who were primarily sedentary. Those classified as field workers, such as drivers, explorers, and mechanical engineers, did outside work. Time of sitting for work with computer/document/meeting was recorded. Shift work for either office or field workers was classified as regular work (8 hours-daytime weekly) and rotating shift work (12 hours-daytime a week and 12 hours-nighttime a week and 2 weeks off). Information regarding the number of daily breaks (not including lunch break) was classified into two groups: 1–2 times/day and more than 2 times/day, and information regarding the duration of break (min/break) and the type of activity performed was also collected.

Recreation was classified by type of activity performed at home consisting of playing with a computer/tablet/smart phone, exercising, and performing other activities. The duration of recreational activity was collected as hours daily. Commute was defined as the mode and duration (h/day) of commuting between home and workplace. The modes of transportation consisted of walk, bicycle, motorcycle, personal car, public transportation, and employee shuttle bus.

This study examined whether personal factors and sedentary behavior was associated with having diseases (4 common NCDs and 2 CMRFs). The risk of having diseases associated with personal factors was determined in order to identify potential confounding factors. We then controlled for these factors to determine the risk of sedentary behavior associated with disease prevalence.

The sample size was estimated using a single proportion formula [20]. The Z statistic of 95% confident level was 1.96, and expected prevalence of diseases (p) was 0.3 [6]. Precision was 20%, and the estimation of prevalence error (d) was 0.03. The required sample size was 1120 subjects after considering a 20% nonresponse rate.

Data were analyzed using SPSS® (version 19.0; IBM, Armonk, NY, USA). The number and percentage (%) of the total population were calculated for categorical data. Mean and standard deviation was used to report the average amount of time spent working, engaging in recreation, and commuting. Total duration of sedentary behavior represented as hours daily (h/day) was calculated by summarizing time spent sitting for work, sitting and playing with computer/tablet/smart phone for recreation, and using a passive mode of transportation when commuting [21]. The prevalence of four NCDs and two CMRFs was calculated and presented as a percentage (%). Cross tabulation and a chi-square test were used to determine whether the prevalence of diseases was associated with personal factors. Age and BMI were confounding factors because they were significantly associated with NCDs and CMRFs. We also considered the frequency of exercise as a confounding factor according to a previous study’s recommendation [5,13,14,15,16,17]. For multivariate analysis, we determined activities and duration of sedentary behavior associated with the occurrence of four NCDs and two CMRFs after adjusting for age, BMI, and exercise. Unadjusted and adjusted odds ratios (ORs) with a 95% confident interval (95% CI) were calculated and analyzed using multiple logistic regression. The association was interpreted as follows: (a) an OR higher than 1.0 indicated a risk factor and was positively associated with diseases, and (b) an OR lower than 1.0 indicated a protective factor and was negatively associated with diseases. The assumption of multiple regression included no multicollinearity (the tolerance >0.1 and VIF < 3) and using a stepwise approach for evaluating a goodness of fit [22]. The significance level was set at *p* < 0.05.

## 3. Results

Of a total of 3365, 1295 subjects (39%) registered for the online questionnaire and 1133 workers (34%) completed the OSCWE. A NCD (*n* = 102), a CMRF (*n* = 237), or a combination (*n* = 66) was reported by 36% of subjects. The majority of subjects selected more than one category of health problem. Subjects reported having one (*n* = 309), two (*n* = 78), three (*n* = 17), and four conditions (*n* = 1). They reported CVDs (*n* = 11), DM (*n* = 24), CRDs (*n* = 129), CAs (*n* = 11), HT (*n* = 82), and hyperlipidemia (*n* = 263). Of 1133, 728 (64%) subjects reported being healthy (*n* = 308) or having other conditions (*n* = 420) such as musculoskeletal disorders, gastrointestinal symptoms, thyroid disease, eyes problems, migraine, stress, and injury.

Our results showed that the majority of subjects were male adults over 30 years with a high BMI (>27.5). They reported a weight change within two years including a weight gain of 3.26 ± 4.02 kg and a weight loss of 5.28 ± 4.81 kg. Only 15% of workers performed frequent exercise (more than 3 times weekly) such as playing sports (golf, football, badminton, swimming), performing yoga, and exercise for physical fitness. Other recreational activities included reading, listening to music, watching TV or movies, knitting, shopping, gardening, and walking their dogs.

The prevalence of diseases among workers was 36% (*n* = 405/1133). Table 1 shows the association between personal factors and disease prevalence. The chance of having diseases approximately doubled in each decade of life after the age of 30 (OR = 1.54–4.28, *p* < 0.05). Workers with high BMI (>27.5) were twice as likely to have diseases compared to those with an acceptable BMI 18.5–23.0 (OR = 2.0, *p* < 0.001). Decreased risk of having diseases was demonstrated in the subjects who reported more frequent exercise in one week.

Table 2 and Table 3 show the association between disease occurrence and sedentary behavior, either at work, during recreation, or while commuting.

More than 39% (*n* = 357/917) of office workers reported having diseases. The odds of having diseases was 3.06 times (95% CI 2.08, 4.49) higher in office workers compared to field workers. The daily workplace sitting time ranged from 5 to 12 h. Those with 12 h rotating shift work were less likely to have diseases than those who had regular work. Of the total, 1030 subjects (91%) reported having breaks daily, and the time ranged from 5 to 30 min for each break. Subjects taking fewer breaks were more likely to have diseases than those who had more breaks. Approximately 80% of the subjects reported that, during breaks, they performed a variety of activities including having coffee while standing or walking (*n* = 695) and sitting or inclining to play with a computer/tablet/smart phone (*n* = 140). Those who were active during breaks were less likely to have diseases compared to those who were not active during break.

The risk of having a disease increased among workers who spent time using a computer/tablet/smart phone at home. Recreation at home was reported by 1124 subjects. There were 535 subjects who reported spending time using a computer/tablet/smart phone and of these 505 subjects spent 1 to 4 h daily. An average of 2 h of sitting for recreation at home was reported by subjects who had and did not have disease (Table 3).

In terms of commute, most subjects 70% used passive modes of transportation including a personal car (*n* = 550), public transportation (*n* = 168), an employee shuttle bus (*n* = 55), or a motorcycle (*n* = 20). The time of transportation between home and work was 15 min–3 h for using passive modes and 15–75 min for walking or bicycling. The subjects who used passive modes to commute were more likely (OR = 1.83, 95% CI 0.93–3.62) to have diseases than those who walked or biked.

After adjusting for age, BMI, and exercise, the risk of having diseases was doubled among workers who engaged in sedentary behavior at work, during recreation, and while commuting. The average total time of sedentary behavior was 10 h/day and was significantly associated with the occurrence of diseases among workers (*p* < 0.001) (Table 3).

## 4. Discussion

Age, BMI, and exercise were confounding factors for the association between sedentary behavior and disease prevalence. Without controlling for these factors, the apparent associations between sedentary behavior and NCD and CMRF occurrence among workers has not been demonstrated [5,13]. By contrast, our findings showed that sedentary behavior at work (OR 2.16, 95% CI 1.52–3.07, *p* < 0.001) and recreation (OR 1.50, 95% CI 1.17–1.90, *p* < 0.001) was associated with NCDs and CMRFs. We found a positive association between sedentary office and shift work, rest during breaks, and sedentary recreation at home and the occurrence of diseases (Table 2). After adjusting for age, BMI, and exercise, the risk of disease associated with workplace sitting time increased by 40%.

Breaking up prolonged sitting periods at work and active commuting benefitted the body’s ability to regain metabolism and stimulate cardiovascular function [16,23]. Workers who took breaks more than twice daily had 41% less risk of NCDs and CMRFs. Those who took more than two active breaks were less likely to have NCDs and CMRFs. Our results correspond to a study by Mailey and colleagues [24]. They reported a decrease in sedentary behavior among workers who took active 1–2 min breaks every half an hour compared to those who had two 15-minute breaks per workday. They collected cardiometabolic markers including total cholesterol, triglycerides, and fasting blood glucose, which were all slightly improved after 8 weeks of intervention. Although our study did not explore this method of breaking up sitting periods, increasing the number of breaks could reduce these periods and promote a more active workplace environment.

The risk of NCDs and CMRFs were reduced 55% among those who used active transport such as walking or cycling. Our findings were consistent with Rissel and colleagues [25]. They used statistical modeling to predict the benefit of increased walking time per day among inactive adults. They proposed that encouraging employees to walk 16 min during their daily commute could promote physical activity if they could not use active transportation.

The average total time of daily sedentary behavior was 10 h/day, and two-thirds of this time occurred in the workplace—8 h of time spent sitting per day. Previous studies have reported that the total time for daily sedentary behavior was 7 h for Dutch workers [26], 10 h for Australian workers [27], and 9 h for East European workers [28]. In these studies, approximately two-thirds of total time spent sitting occurred at work (4.0–6.0 h). However, they did not assess total daily accumulated time spent sitting on health risk. Our findings demonstrated that time spent sitting at work and total sedentary behavior were associated with disease occurrence after adjusting for age, BMI, and exercise (*p* = 0.001). Prolonged sitting time at work could be used as a predicting factor to determine whether workers are at a high risk of disease occurrence. Recent findings by Pronk and colleagues [29] reported positive correlations between sedentary behavior at work and musculoskeletal pain, fatigue, and emotion (*r* = 0.35–0.47, *p* < 0.05). They found that stand-up projects during sedentary work could reduce 66 min of time spent sitting and improve neck and back musculoskeletal pain, fatigue, mood, and depression.

One unexpected finding in this study was the lower risk of diseases for shift workers after adjusting for age, BMI, and exercise. Previous studies found a low level of physical activity (PA) and a high level of sedentary behavior among shift workers compared with regular workers [30,31]. Day and night rotating shift work disrupted the circadian sleep cycle impacting hormonal and sympathetic responses, resulting in CVDs [31]. These conflicting findings may be due to the different types of work assessed in these studies, so the influence of shift work on health risk needs further investigation.

The negative health risk effect of sedentary behavior could be alleviated by PA [32,33]. Bakrania and colleagues [33] examined the interaction effect of PA and sedentary behavior influencing the markers of cardiometabolic health among British adults 18 years and over. They reported a low BMI, waist circumference, glycated hemoglobin (HbA1c) levels, and high HDL-cholesterol levels among adults who were physically active compared to those with low to high sedentary status (*p* < 0.01). Ekelund and colleagues [34] determined whether sedentary behavior and PA were associated with all-cause mortality. The meta-analysis findings demonstrated that performing moderate intensity of PA (i.e., 60–75 min/day) could lower the risk of dying for those who sat more than 8 h/day. They concluded that all-cause mortality risk resulting from prolonged sitting could be eliminated by PA. Our recommendations are similar. Interrupting prolonged sitting while working, and having active leisure time and commuting, were beneficial. The total time of sedentary behavior of our workers did not significantly differ among the four groups of exercise, but a low risk of diseases was found among workers who performed exercise during the week compared with those who did not. 

Some limitations occurred in this study. First, we conducted a cross-sectional survey that might not demonstrate causality between sedentary behavior and disease occurrence among workers. However, we provided strong evidence of a positive association by eliminating the effect of confounding factors. We filled in the gap of controversial relationships and recommendations for workers. Second, the low questionnaire response rate (34%) might not have represented all workers. However, the number of participants was greater than the sample size calculation. Statistical significance at a *p*-value less than 0.05 and 0.001 was demonstrated in many parameters in our study. Third, the self-reporting questionnaire could have resulted in recall or information bias. These problems are inevitable in survey-based research. However, we minimized these problems by using an online self-reporting questionnaire that was user-friendly, took only 20 min to administer, and was appropriate for surveying health risk factors among workers.

## 5. Conclusions

In conclusion, we found positive associations between sedentary behavior and four common NCDs as well as two CMRFs among workers. Based on our findings, recommendations to prevent health risk were developed. First, workers should reduce time spent sitting at work by arranging short and more frequent active breaks. Second, workers should avoid prolonged sitting during commuting by walking or cycling. Third, workers should be conscious of performing more PA or exercise in their spare time than inactive or sedentary recreation. Finally, an organization should implement strategies to promote PA to compensate for prolonged sedentary behavior while working such as stand-up projects or active work stations.

## Figures and Tables

**Table 1:** Association between personal factors and disease prevalence (specifically CVDs, DM, CRDs, CAs, HT, and hyperlipidemia) among 1133 workers in Thailand.

Characteristics	Total	Diseases	Prevalence Rate (95%)	OR	95% CI		*p*-Value
					Lower	Upper	
Age (years), *n* = 1133							
20–29	349	87	24.9	1.00	-	-	-
30–39	475	161	33.9	1.54	1.14	2.10	0.006 *
40–49	205	96	46.8	2.65	1.84	3.83	<0.001 **
50–62	104	61	58.7	4.28	2.70	6.76	<0.001 **
Sex, *n* = 1133							
Male	613	227	37.0	1.00	-	-	-
Female	520	178	34.2	1.13	0.89	1.44	0.33
Body Mass Index (BMI), *n* = 1133							
BMI < 18.5	98	28	28.6	0.87	0.54	1.40	0.56
18.5 ≤ BMI ≤ 23	475	150	31.6	1.00	-	-	-
23 < BMI ≤ 27.5	404	152	37.6	1.31	0.99	1.73	0.06
BMI > 27.5	156	75	48.1	2.06	1.43	2.95	<0.001 **
Weight change in the last 2 years, *n* = 1128							
Weight stable	406	139	34.2	1.00	-	-	-
Weight gain	549	201	36.6	1.11	0.85	1.45	0.449
Weight loss	173	65	37.6	1.16	0.80	1.67	0.442
Year of work experience, *n* = 1126							
Less than and 3 years	490	179	36.5	1.00	-	-	-
4 to 6 years	184	64	34.8	0.93	0.65	1.32	0.674
7 to 10 years	246	91	37.0	1.02	0.74	1.40	0.903
Over 10 years	206	68	33.0	0.86	0.61	1.21	0.376
Exercise, *n* = 1124							
No exercise	212	79	37.3	1.00	-	-	-
Less than and once/week	417	147	35.3	0.92	0.65	1.29	0.619
2 to 3 times/week	315	116	36.8	0.98	0.68	1.40	0.919
Over 3 times/week	180	62	34.4	0.86	0.58	1.34	0.562

“Diseases” refers to four common NCDs and two CMRFs (CVDs, DM, CRDs, CAs, HT, and hyperlipidemia); * *p*-value < 0.05, ** *p*-value < 0.001.

**Table 2:** Association between the sedentary behaviors and diseases among workers (for categorical variables).

Sedentary Behaviors	Diseases	Non-Diseases	Unadjusted			Adjusted by Age, BMI and Exercise		
			OR	95% CI	*p*-Value	OR	95% CI	*p*-Value
Work								
Job description (*n* = 1123)								
Office work	357	560	2.16	1.52, 3.07	<0.001 *	3.06	2.08, 4.49	<0.001 **
Field work	47	159	1.00	-	-	-	-	-
Shift work (*n* = 1099)								
Regular work	361	614	1.84	1.19, 2.83	0.005 *	2.55	1.60, 4.06	<0.001 **
12-h shift work	30	94	1.00	-	-	-	-	-
Rest of break (*n* = 1030)								
Less than and twice/day	346	559	1.53	1.02, 2.31	0.042 *	1.70	1.11, 2.60	0.015 *
More than twice/day	36	89	1.00	-	-	-	-	-
Recreation at home (*n* = 1124)								
Spending time using computer/tablet/smart phone								
Yes	218	317	1.50	1.17, 1.90	0.001	1.99	1.52, 2.59	<0.001 **
No	186	403	1.00	-	-	-	-	-
Commute (*n* = 839)								
Passive mode ^a^	330	466	1.83	0.93, 3.62	0.082	2.21	1.10, 4.57	0.033 *
Walking/cycling	12	31	1.00	-	-	-	-	-

^a^ Passive mode of transportation included personal car, motorcycle, public transportation, and employee shuttle bus. “Diseases” refers to four common NCDs and two CMRFs (CVDs, DM, CRDs, CAs, HT, and hyperlipidemia); * *p*-value < 0.05, ** *p*-value < 0.001.

**Table 3:** Association between the sedentary behaviors and diseases among workers (for continuous variables).

Time (Hour/Day)	Diseases		Non-Diseases		Unadjusted			Adjusted by Age, BMI and Exercise		
	*n*	Mean ± SD	*n*	Mean ± SD	OR	95% CI	*p*-Value	OR	95% CI	*p*-Value
Workplace sitting	381	8.20 ± 2.41	653	7.95 ± 2.50	1.04	0.99, 1.10	0.123	1.10	1.04, 1.16	0.001 *
Spending time using computer/tablet/smart phone for recreation at home	210	2.83 ± 1.03	296	2.84 ± 1.02	1.00	0.83, 1.18	0.920	1.04	0.87, 1.25	0.640
Passive mode of transportation	330	1.78 ± 1.62	464	1.70 ± 0.97	1.07	0.96, 1.20	0.202	1.06	0.95, 1.18	0.309
Total time of sedentary behavior	380	11.18 ± 3.77	656	10.43 ± 3.99	1.05	1.02, 1.09	0.003 *	1.10	1.06, 1.14	<0.001 **

“Diseases” refers to four common NCDs and two CMRFs (CVDs, DM, CRDs, CAs, HT and hyperlipidemia); * *p*-value < 0.05, ** *p*-value < 0.001.
